# Current Evidence for IL-17/23 Blockade for the Treatment of Lupus Nephritis

**DOI:** 10.7759/cureus.20087

**Published:** 2021-12-01

**Authors:** Juan Camilo Santacruz, Sandra Pulido, Angelo Arzuaga, Marta Juliana Mantilla, Ana María Santos, John Londono

**Affiliations:** 1 Spondyloarthropathies Research Group, Universidad de La Sabana, Chía, COL; 2 Rheumatology Department, Universidad Militar Nueva Granada, Bogotá, COL

**Keywords:** biological therapy, ustekinumab, secukinumab, lupus nephritis, il-17 inhibitor therapy

## Abstract

In the current medical literature, there is increasing evidence for the involvement of the interleukin (IL)-17/23 axis and the potential role of Th17 cells in the pathogenesis of lupus nephritis. Knowledge about the interaction of these immunological pathways in the development of autoimmune diseases has led to the identification of new therapeutic strategies aimed at blocking them. For this reason, the main objective of this review focuses on knowing the recent evidence of the different anti-IL-17/23 treatment strategies in lupus nephritis and their future perspectives. A non-systematic narrative review of the literature was carried out following the objective of having the most representative information on the different anti-IL-17/23 drugs available together with the description of the pathophysiological mechanisms of this pathway involved in systemic lupus erythematosus and lupus nephritis. Despite the great existing theoretical foundation, today few clinical studies support the use of these therapies in both contexts. Nevertheless, the publication of research with a better methodology is expected to approve the indication of some of these drugs in lupus nephritis. However, the clinical response seen with ustekinumab and secukinumab in clinical studies and case reports published to date has been encouraging.

## Introduction and background

Lupus nephritis (LN) is the most common severe organic manifestation of systemic lupus erythematosus (SLE), rapidly progressing to end-stage renal failure unless promptly treated [1]. Renal biopsy remains the gold standard as it provides important information about LN classification, the severity of kidney injury, pathologic activity, and chronicity [2]. Currently, the classification of LN continues to be closely related to the therapeutic response and long-term renal outcome [3]. In recent years, great advances have been made in understanding the pathophysiology of LN by proposing different therapeutic approaches to prevent progression to end-stage renal failure. Today, multiple immunological pathways involved in the induction of tissue damage in SLE have been described. It should be noted that cytokines are fundamental mediators in this process and, among them, the role of the interleukin IL-17 / IL-23 axis and Th17 cells have recently emerged, which appear to play an important role in the pathogenesis of LN [4]. Patients with SLE are known to have elevated levels of IL-17 in serum, and increased expression of this cytokine has also been observed in compromising target tissues [5]. Furthermore, it has been shown that IL-23 levels are elevated in peripheral blood mononuclear cells, achieving IL-17A induction by co-stimulated leukocytes with IL-23 in patients with LN [6]. Th17 cells are involved in several pathological pathways of SLE, such as the induction of vascular inflammation, the recruitment of leukocytes, the activation of B cells, and the production of autoantibodies, ultimately contributing to glomerular injury and persistence of inflammation [7]. Today, despite the great theoretical foundation for blocking the interleukin IL-17/IL-23 axis as a promising therapeutic target, there are no controlled clinical studies that support its use in daily clinical practice in LN. This review focuses on describing the available and ongoing treatments for interleukin IL-17/IL-23 axis blockade as a treatment option for lupus LN and refractory LN.

## Review

Methods

**Figure 1:**
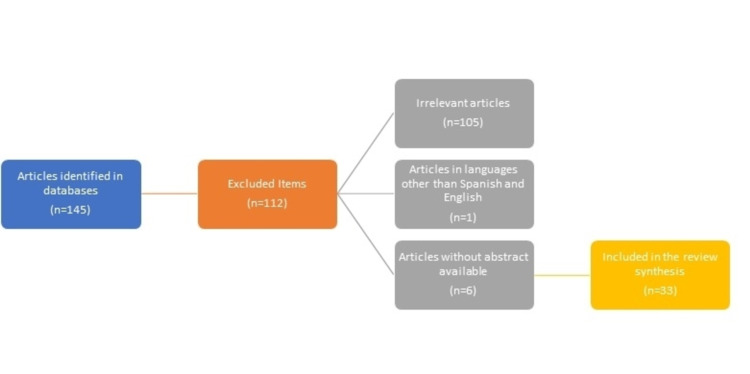
Search diagram

A non-systematic narrative review of the literature developed in English and Spanish was carried out, following the objective of having the most representative information for the referenced articles until 2021 in primary databases such as Pubmed, Embase, and Lilacs. The MESH (medical subject headings) terms used were: "IL-23", "IL-17", "systemic lupus erythematosus", "therapeutic antibodies", and "lupus nephritis" combining boolean operators (AND, OR). Below is a flow chart detailing the search strategy (Figure 1).

Role of the interleukin (IL)-23/IL-17 axis in the pathophysiology of SLE

**Figure 2:**
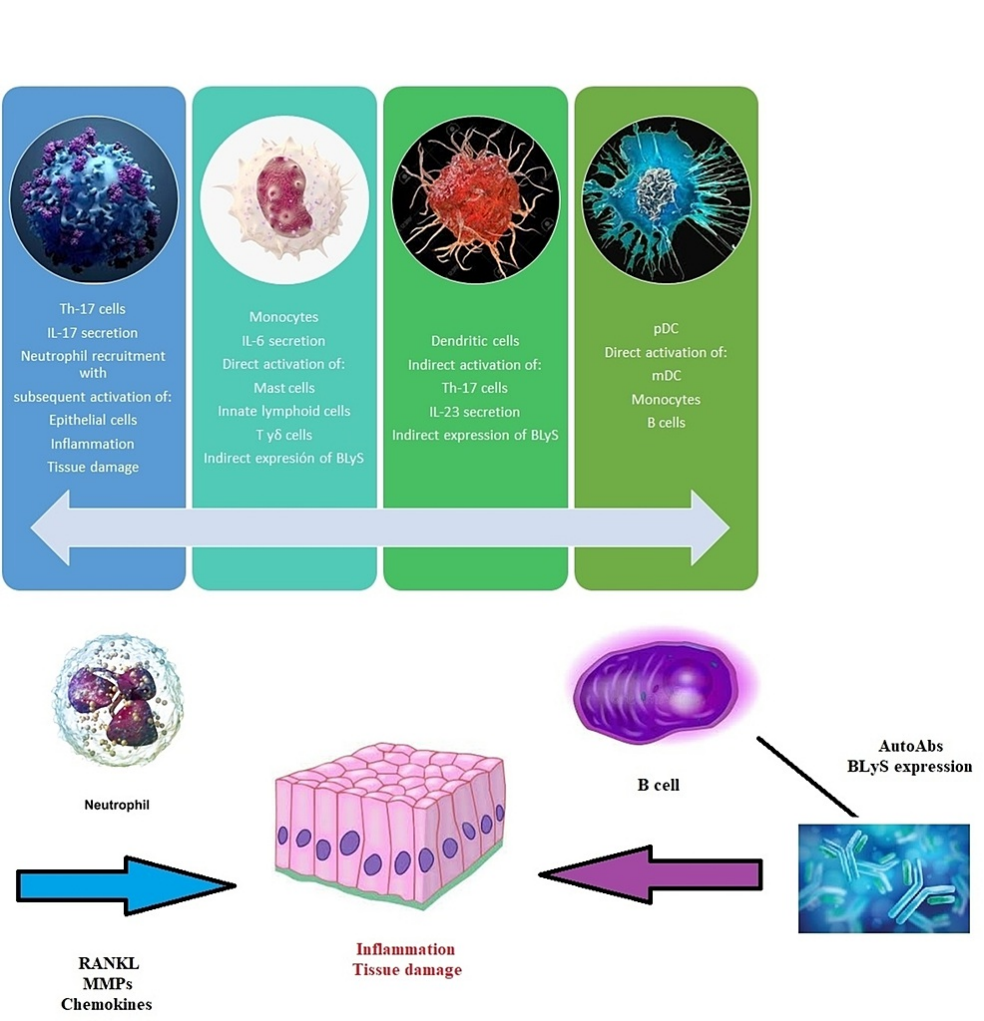
Synopsis of the pathophysiology of the IL-17/23 axis and its involvement in SLE AutoAbs: autoantibodies; BLys: B-cell activating factor; mDC: myeloid dendritic cells; MMPs: Matrix metalloproteinase; pDC: plasmacytoid dendritic cells; RANKL: receptor activator of nuclear factor κB ligand; SLE: systemic lupus erythematosus; IL: interleukin

The initial steps in the differentiation of naive CD4 + T cells into IL-17 producing cells do not require IL-23. However, IL-23 plays an important role in stabilizing the phenotypic characteristics of the T helper (Th)17 lineage. IL-23 is important in the expansion and maintenance of Th17 cells, acting mainly on effector T helper cells and memory CD4 + cells to enhance IL-17 secretion [8]. The production of IL-12 and IL-23 requires nuclear factor kappa B (NF-κB), which triggers initial immune responses that lead to Th1 or Th17 cell-mediated immunity. Th17 cells differentiate from naive T cells under the influence of transforming growth factor beta TGF-β) and IL-6, but their maintenance and expansion are mainly mediated by IL-23 [9]. Without IL-23, CD4 + cells in the presence of these cytokines can produce large amounts of IL-17 but do not fully develop into pathogenic Th17 cells [10]. In mice prone to SLE, IL-23 receptor deficiency was shown to reduce IL-17 production, being a protective factor for disease initiation [11]. The IL-17 / IL-23 axis is involved in the pathogenesis of SLE when activated dendritic cells (DC) produce inflammatory cytokines (IL-6 and IL-23), stimulating Th17 cells to produce IL-17. Furthermore, since IL-17 has an important role in the genesis and progression of kidney disease, it has been proposed as a potential biomarker [12]. High levels of interferon (INF)-α produced by plasmacytoid dendritic cells (CDp) have also been reported to promote the activation of antigen-presenting cells that also activate Th17 cells to produce IL-17 [13]. Activated monocytes induce IL-17 production by three cell groups (innate lymphoid cells, T and δ cells, and mast cells), in addition to producing IL-6 and IL-23, which also trigger Th17 cell activation. Recently, higher frequencies of circulating Th17 cells have been confirmed in patients with class IV and V LN, correlating even with higher Systemic Lupus Erythematosus Disease Activity Index (SLEDAI) scores [14]. On the other hand, there is growing evidence that IL-6 can improve Th17 cell differentiation by promoting the sequential participation of IL-21/IL-23 pathways, which play a critical role in the genesis of autoimmune diseases [15]. Additionally, there is a greater glomerular expression of IL-17, IL-6, IL-18, and IL-23 in patients with class IV LN compared to healthy controls, which supports that there is a greater infiltration of Th17 cells in the glomeruli and a higher expression of urinary cytokines derived from this signaling pathway [16, 17]. Finally, the production of autoantibodies by activated B cells leads to the activation of DC to secrete IL-23, which also contributes to the increased production of IL-17 [18]. IL-17 induces the production of a wide variety of inflammatory cytokines, receptor activator of nuclear factor κB ligand (RANKL), matrix metalloproteinases, and chemokines, resulting in the recruitment of neutrophils that mediate inflammation and tissue damage (Figure 2) [19]. 

Biological drugs implicated in the blockade of the IL-17 and IL-23 axis

IL-17 comprises a family of six homologous cytokines (IL-17A to F), of which IL-17A is the most abundant, most potent, and best characterized. The IL-17 signal is translated through dimeric receptors, of which there are five identified subunits (IL-17 RA to E) [20]. Secukinumab is a fully humanized monoclonal antibody against IL-17A, and it is effective in the treatment of plaque psoriasis, psoriatic arthritis, and ankylosing spondylitis [21]. Ixekizumab is a humanized anti-IL-17A antibody that has shown similar efficacy for psoriasis and psoriatic arthritis [22]. Brodalumab is a humanized monoclonal antibody against IL-17RA, necessary for the formation of dimeric receptors for the signaling of IL-17A, IL-17F, IL-17A/F, IL-17C and IL-17E [23]. It is approved for the treatment of psoriasis in Japan and the United States. Ustekinumab is a fully humanized IgG1k monoclonal antibody that binds to the p40 subunit to inhibit both IL-12 and IL-23, preventing them from binding to its receptors on the surface of immune cells [24]. The antibody interferes with the activities of the Th1-Th17 pathways and also in the activation of keratinocytes [25]. By binding to this subunit, it prevents binding to the cell surface receptor IL-12Rb1, which hinders the activity of both cytokines. Ustekinumab has been approved for psoriasis, psoriatic arthritis, and Crohn's disease [26]. Next, a section is made with the most promising drugs in progress with this class of drugs for the treatment of SLE and the evidence that exists in LN.

Ustekinumab

The safety and efficacy of ustekinumab in patients with active SLE were evaluated in a phase II study. A placebo-controlled trial was conducted in patients with seropositive SLE (anti-nuclear antibody [ANA], double-stranded DNA [dsDNA] and/or anti-Smith antibodies) and patients who had and active disease (SLEDAI ≥6, British Isles Lupus Assessment Group index [BILAG] A ≥1 and/or ≥2 BILAG B scores) despite standard therapy. Patients were randomized (3:2) to receive intravenous ustekinumab (~ 6 mg/kg) or placebo followed by subcutaneous injections of ustekinumab 90 mg in the intervention group. Ustekinumab conferred significantly better efficacy compared to placebo, where 60% of patients receiving ustekinumab showed an improvement in SLE response rate (SRI-4) versus 31% in the placebo group after 24 weeks. The risk of a new flare from BILAG was significantly lower than placebo (p = 0.0078). In addition, it demonstrated an improvement in mucocutaneous and musculoskeletal disease, as well as a decrease in anti-dsDNA titers and low C3 levels [27]. There are no clinical cases reported for the use of ustekinumab in LN and refractory LN.

Secukinumab

A phase III, randomized, double-blind, parallel-group, placebo-controlled, 2-year study is currently underway to evaluate the efficacy and safety of secukinumab in combination with standard therapy in patients with active lupus nephritis (NCT04181762). The primary endpoint is the proportion of patients who will achieve a complete renal response [28]. There are very few reported cases of secukinumab for the treatment of LN and refractory LN. Among the most representative cases is that of a 62-year-old woman who presented plaque psoriasis and refractory LN for which she was treated with secukinumab given the resistance to conventional treatment. The proliferation of activated Th17 cells was confirmed by flow cytometry and the infiltration of numerous IL-17 positive lymphocytes into the interstitium was demonstrated in kidney tissue. After the initiation of secukinumab, the clinical and biological characteristics improved [29]. The other case described describes the case of a woman diagnosed with SLE since 2011 with joint and skin involvement undergoing treatment with 10 mg of prednisolone and 400 mg of hydroxychloroquine, achieving stability of the symptoms for a short time. Later treatment with methotrexate was indicated, but due to recurrence of joint flare-ups and persistent activity, treatment with rituximab was indicated with substantial improvement. In 2017, she presented a nephritic syndrome with class IV renal biopsy requiring induction with mycophenolate and a new cycle of rituximab obtained only partial remission, requiring the additional administration of immunoglobulin and belimumab. In 2019, due to the persistence of cutaneous involvement and presenting renal relapse, secukinumab (300 mg sc monthly) was indicated, achieving complete remission [30].

IL-17 and IL-23 as predictors of response to treatment

It has been suggested that IL-17 and IL-23 can be alternative biomarkers for the diagnosis, monitoring, activity, and prediction of the response to treatment in LN. Measurement of these specific cytokines has even been considered to assess the severity of the disease and to replace renal biopsy at some point [31]. IL-17 and IL-23 levels have also been shown to decrease significantly in patients with active LN who are undergoing treatment. This has been demonstrated by a study carried out in China in which 80 patients with LN and 20 healthy controls were recruited by measuring baseline plasma levels of IL-17 and IL-23 by enzyme-linked immunosorbent assay (ELISA) technique, wherein the end 37 patients with active LN accepted immunosuppressive therapy with a 6-month follow-up. Baseline IL-17 and IL-23 levels were higher in patients with active LN compared to controls. At 6 months, IL-17 and IL-23 levels decreased significantly in patients with active LN after treatment [32]. Another study demonstrated the association of IL-17 and IL-23 levels with histopathological findings and response to treatment, including 52 patients with active LN, performing renal biopsies at the beginning and after immunosuppressive treatment. Serum levels of tumor necrosis factor (TNF)-α, IFN-γ, IL-6, IL-10, IL-17, IL-23, and TGF-β were analyzed at both times during the biopsy and were also measured in 13 healthy controls. IL-17 expression in kidney tissue was evaluated by immunohistochemistry. Biopsies were evaluated against the WHO classification and kidney disease activity was estimated using the BILAG index. The improvement in 2 degrees in the renal BILAG was considered as a complete response and in 1 degree as a partial response. At the beginning of the study, all patients had high disease activity (BILAG A/B). Baseline IL-17 levels were higher in patients with persistent active nephritis (WHO III, IV, V) after treatment. At follow-up, IL-23 was higher in those who did not achieve improvement in BILAG. It was concluded that a high baseline IL-17 predicted an unfavorable histopathological response, and those who did not achieve an improvement in BILAG had higher levels of IL-23, indicating that a subset of patients with LN has a Th-17 phenotype that may influence the response to treatment and could be evaluated as a biomarker of poor therapeutic response [33]. The clinical studies that represent the strongest evidence for IL-17/23 axis block for the treatment of LN are presented below in Table 1.

**Table 1:** Published and ongoing clinical studies of IL-17/23 axis block for the treatment of LN Sc: subcutaneous; SRI4: lupus-4 response index; LN: lupus nephritis

Name and study drug	Type of study	Number of patients	Comment	Reference
STELARA (Ustekinumab)	Intervention	102	To assess the response of ustekinumab using SRI-4 and its safety compared to placebo	[27]
AIN457 (Secukinumab)	Intervención	~460	To evaluate the efficacy and safety of secukinumab (300 mg Sc) compared to placebo, in patients with active LN	[28]

## Conclusions

At present there is a great foundation to consider the importance of the IL-17/23 axis in the pathogenesis of LN, considering both cytokines as biomarkers of progression, disease activity and response to treatment. Despite this, there is a great limitation regarding the ongoing studies with the different molecules available to block this pathway. Ustekinumab could be a potential strategy for the treatment of active SLE and LN. Although the results of the ongoing phase III study with secukinumab are awaited to approve its indication in LN, the clinical response that has been observed in the cases published to date is encouraging. Brodalumab and ixekizumab do not have ongoing clinical studies for the treatment of LN, however, it is not ruled out that they may be the subject of future research. More clinical studies are required to consider IL-17/23 axis block as a treatment option for LN, although the evidence is growing and more promising every day.
